# Functional Analyses Reveal Extensive RRE Plasticity in Primary HIV-1 Sequences Selected under Selective Pressure

**DOI:** 10.1371/journal.pone.0106299

**Published:** 2014-08-29

**Authors:** Francesc Cunyat, Nancy Beerens, Elisabet García, Bonaventura Clotet, Jørgen Kjems, Cecilia Cabrera

**Affiliations:** 1 IrsiCaixa-HIVACAT, Institut de Recerca en Ciències de la Salut Germans Trias i Pujol (IGTP), Hospital Germans Trias, Universitat Autònoma de Barcelona, Badalona, Barcelona, Catalonia, Spain; 2 Department of Molecular Biology, Aarhus University, Aarhus, Denmark; 3 Interdisciplinary Nanoscience Center (iNANO), Molecular Biology and Genetics Department, Aarhus University, Aarhus, Denmark; International Centre for Genetic Engineering and Biotechnology, Italy

## Abstract

**Background:**

HIV-1 Rev response element (RRE) is a functional region of viral RNA lying immediately downstream to the junction of gp120 and gp41 in the *env* coding sequence. The RRE is essential for HIV replication and binds with the Rev protein to facilitate the export of viral mRNA from nucleus to cytoplasm. It has been suggested that changes in the predicted secondary structure of primary RRE sequences impact the function of the RREs; however, functional assays have not yet been performed. The aim of this study was to characterize the genetic, structural and functional variation in the RRE primary sequences selected *in vivo* by Enfuvirtide pressure.

**Results:**

Multiple RRE variants were obtained from viruses isolated from patients who failed an Enfuvirtide-containing regimen. Different alterations were observed in the predicted RRE secondary structures, with the abrogation of the primary Rev binding site in one of the variants. In spite of this, most of the RRE variants were able to bind Rev and promote the cytoplasmic export of the viral mRNAs with equivalent efficiency in a cell-based assay. Only RRE45 and RRE40-45 showed an impaired ability to bind Rev in a gel-shift binding assay. Unexpectedly, this impairment was not reflected in functional capacity when RNA export was evaluated using a reporter assay, or during virus replication in lymphoid cells, suggesting that *in vivo* the RRE would be highly malleable.

**Conclusions:**

The Rev-RRE functionality is unaffected in RRE variants selected in patients failing an ENF-containing regimen. Our data show that the current understanding of the Rev-RRE complex structure does not suffice and fails to rationally predict the function of naturally occurring RRE mutants. Therefore, this data should be taken into account in the development of antiviral agents that target the RRE-Rev complex.

## Introduction

Introns in mRNAs are generally spliced before leaving the nucleus in mammalian cells. However, retroviral replication requires the export of some viral RNAs with one or more of their introns retained. HIV-1 fulfills this requirement by encoding the Rev protein, which is a posttranscriptional regulator that plays an essential role in virus replication (reviewed in reference [1]). One well-defined function of Rev is to bind to the Rev response element (RRE), present in all unspliced and incompletely spliced RNAs, to promote their nucleo-cytoplasmic export [2]–[4]. Nevertheless, Rev is also reported to play more roles in the retrovirus life cycle, including: polyadenylation, RNA splicing, RNA stability, translation and encapsidation (reviewed in [5]).

The RRE encompasses a ≈328-nucleotide RNA segment located within the junction of the gp120- and gp41-encoding sequences [1], [3]. It adopts an extensive secondary structure including a series of five stems (I, II, III IV and V) arranged around a central junction, with stem-loop II split into a proximal stem (IIA) and two distal stem-loops (IIB and IIC) situated around a three-way junction [6], [7]. There is a high-affinity site for Rev in stem-loop IIB [2]–[4], [8], [9] and a second Rev-binding site in stem I [10]. The RRE serves as a scaffold for the cooperative assembly of multiple Rev molecules [2]. Initial occupancy of the IIB site by Rev causes a conformational change [11] that is followed by the binding of additional monomers to the complex, with a correlation between the degree of oligomerization and the ability of Rev to transport the RNA [12].

The relationship among RRE functionality and RRE structure has been evaluated, suggesting stem-loop II as the only region required for Rev function [13]. However, different studies highlighted the importance of other regions throughout the RRE, pointing out that the overall RNA secondary structure is a major determinant for the Rev-RRE interaction and function [2], [4], [8], [14], [15]. Furthermore, a recent study has shown that, in addition to the secondary structure, the RRE must adopt an “A”-like three-dimensional structure for an optimal function [16]. However, in these studies the majority of sequences used were derived from laboratory-adapted HIV strains. This is important because, in HIV infected patients, the RRE has been shown to be structurally variable [17], [18]. Particularly, changes selected in this region over the course of the infection have been associated with modulation in the Rev-RRE activity, which could control viral replication and pathogenesis [19]–[22].

The Rev-RRE activity plays an essential role in HIV-1 replication and makes the disruption of its interactions an attractive target to design effective antiviral therapies. The Rev-RRE activity may be highly refractory to the evolution of resistance because RRE serves both as the high-affinity Rev binding site and forms a part of the open reading frame of the gp41 protein, imposing additional constrains on the evolutionary pathways. *In vitro* studies have revealed the appearance of functional RRE variants with altered secondary structures in cells treated with a Rev variant (REVM10) [23], or with heterocyclic compounds that inhibit Rev-RRE function [24]. This suggests that the sequence and the structure of the RRE can undergo changes under selective pressure without affecting function. Nevertheless, the *in vivo* evolutionary malleability of the RRE under the selective pressure exerted by antiretroviral therapy is still unknown; there are no drugs available for clinical use that target the Rev-RRE interaction.

Enfuvirtide (ENF, T-20) is the first peptide approved for clinical use and represents a new class of drugs known as fusion inhibitors. These inhibitors prevent HIV-1 from binding to and entering the human cell. ENF binds to gp41, and thereby inhibits the conformational change of gp41 that is necessary for fusion of the envelope protein to host cells. Unfortunately, treatment of HIV-1 patients with ENF leads to resistance to the inhibitor. Interestingly, all mutations in gp41 associated with ENF resistance, amino acids 36 to 45, are localized within RRE sequence (in stems IIC, IIA and III) [25], [26]. The emergence of these mutations not only affects the gp41 protein, but could also impact the RRE element, providing a unique opportunity to evaluate the *in vivo* evolution of the RRE functionality under selective pressure.

The objective of the current study was to characterize the sequence, structural and functional variation of the RRE region in HIV-1 infected patients who experienced virologic failure on an ENF-containing regimen. Selected RRE variants showed a high structural variability, resulting in many changes that included the elimination of stem IIA or the formation of stem III. The Rev-RRE function was extensively characterized, revealing that changes at position 45 resulted in a slightly decreased ability to bind Rev. We observed no functional impairment in the RRE variants studied, regardless of the sequence or the predicted secondary structure presented. Our results suggest that the RRE is a flexible structure and the predicted changes in secondary structure of naturally occurring HIV-1 RRE variants may not directly reflect the function of the Rev-RRE pathway.

## Methods

### Cells and plasmids

The 293T cell line was obtained from the American Type Culture Collection (ATCC, LGC Standards, Middlesex, UK) and were grown in Dulbecco's modified Eagle's medium (DMEM) supplemented with 10% of heat-inactivated fetal bovine serum (FBS) and maintained at 37°C in a 5% CO_2_ incubator. MT-4 cells were obtained through the NIH AIDS Reagent Program, Division of AIDS, NIAID, NIH from Dr. Douglas Richman and were grown in RPMI medium with 10% of FBS and appropriate antibiotics. HIV-1 NL4-3 3′ Clone (p83-10) and HIV-1 NL4-3 5′ Clone (p83-2) were obtained through the NIH AIDS Reagent Program, Division of AIDS, NIAID, NIH from Dr. Ronald Desrosiers [27]. pCMV-Rev was obtained through the NIH AIDS Reagent Program, Division of AIDS, NIAID, NIH from Dr. Marie-Louise Hammarskjöld and Dr. David Rekosh [28].

### Clinical samples and ethics statement

Two previously characterized heavily pre-treated HIV-1-infected patients that received an ENF-containing salvage therapy were selected [25]. Plasma samples at different time points during the treatment from patient 5 (who harbored viruses with amino acid changes at positions 36, 38 and 43 in gp41) and patient 10 (with changes at positions 40 and 45) were used to obtain different patient-derived RRE variants. The ethics committee and the institutional review board from the Hospital Universitario Germans Trias i Pujol approved the study and all subjects provided written informed consent.

### Full-length Envelope-expressing plasmids

Plasma samples were used to generate Envelope-expressing plasmids. Viral RNA was isolated (QIAamp Viral RNA kit, QIAgen, Spain) and a fragment corresponding to the rev, vpu and env genes was amplified using the NLEcoRIF and NLXhoIR primers (nucleotides 5284–5310 and 9055–9027 in the HIV _HXB2_ numbering system, respectively) and the RNA-NestedF and the RNA-NestedR primers in a nested PCR (nucleotides 5954–5983 and 8904–8882 in the HIV _HXB2_ numbering system, respectively). The PCR fragment was purified (SNAP UV-free gel purification kit, Invitrogen) and subsequently, directionally cloned into the expression vector pcDNA.3.1D/V5/His-TOPO (Invitrogen). Between 10 and 15 recombinant expression plasmids were obtained from each patient and the envelope region was fully sequenced using specific primers with the Big Dye Terminator v3.1 cycle sequencing kit and the ABI 3100 sequence analyzer (Applied Biosystems, Foster City, California, USA). All sequences were aligned and edited using the programs Sequencher v4.7 (Gene Codes Corporation, Ann Arbor, MI) and GeneDoc v2.6. Full-length envelope clones were classified and used depending on their mutations. The regions corresponding to the first and the second exons of Rev were also sequenced in some of the plasmids.

### RNA secondary structure analysis

The sequence between nucleotides 7730–8058 (HXB2 numbering system) of the obtained clones was used to predict the secondary structure of the RRE using bioinformatic tools. Two different bioinformatics programs, the Vienna RNA Fold computer program (RNAfold) (http://rna.tbi.univie.ac.at/cgi-bin/RNAfold.cgi, [29]) and Mfold (http://mfold.rna.albany.edu/?q=mfold/RNA-Folding-Form [30]), were used to model the RRE secondary structure of the full sequence of our RRE variants in order to evaluate the ability of the present nucleotides to induce conformational changes in the RRE.

### Site-directed mutants

RRE-expressing plasmids that contained Q40H, L45M and 40Q-45L were generated by site-directed mutagenesis, using the GeneTailor Site-Directed Mutagenesis Kit (Invitrogen). Site-directed mutants were created using as a template a full Env-expressing plasmid that was derived from patient 5 and which harbored the double mutation RRE40-45 (Q40H-L45M). The RRE fragments generated after the site-directed mutagenesis were subsequently cloned into the pPCR-Script Amp SK(+) cloning vector to test the Rev-RRE binding or into the pDM628ΔRRE to test the Rev-dependent transport and sequenced, as described below.

### RRE-expressing plasmid for *in vitro* RNA production

RRE-expressing plasmids for *in vitro* RNA synthesis were constructed from the full-length Env-expressing plasmids. The fragment that corresponded to the RRE region (328 bp) was PCR-amplified using the RREF and RRER primers (nucleotides 7730–7749 and 8058–8041 in the HIV _HXB2_ numbering system, respectively), purified by gel-excision (SNAP UV-free gel purification kit) and cloned into the pPCR-Script Amp SK(+) cloning vector (Cultek, Granollers, Spain) containing the T7 promoter, as described by the manufacturer. Several clones were selected and sequenced to confirm the presence of the changes of interest and to discard the presence of new nucleotide changes.

### *In vitro* synthesis of RRE RNA and electrophoretic mobility shift assay (EMSA)

RRE RNA was generated through *in vitro* transcription by T7 RNA polymerase and radiolabeled UTPs from the constructed RRE-expressing plasmids after enzymatic digestion of the plasmid. The RNA products were separated on a denaturing gel and the full-length products were isolated. After elution, the RNA was precipitated and the percentage of ^32^P-UTP incorporation was determined. Equal amounts of RNA were used for Rev binding assays. Recombinant Rev protein was produced in *E.coli*, purified, and then mixed in varying concentrations with the RRE RNA. The products were analyzed on a native 4% polyacrylamide gel. The products were quantified by phosphoimager scanning. The percentage shift was calculated by dividing the amount of shift by the total amount of RNA (shift/(RRE + shift))*100%.

### RRE-expressing plasmid to measure RNA transport

The pDM628ΔRRE vector (with a deletion of 481 bp) was constructed by introducing into the reporter vector pDM628, a Rev-dependent luciferase-based vector [31], [32], two cleavage sites for two restriction enzymes at the 5′ and 3′ ends of the RRE region using PCR based mutagenesis. The enzymatic cleavage sites were introduced by inverse PCR (primers: SpeI-pDMRREF TTAACAATTACACTAGTTTAATACACTCC and XmaI-pDMRRER TATCTCCTCCCCCGGGTCTGAAGA, nucleotides 8128–8156 and 7647–7624 in the HIV _HXB2_ numbering system, respectively). The PCR fragment obtained was digested with *SpeI* and *XmaI* (New England BioLabs) and the polylinker 5'PO4 CCGGGACCGGTGGCGCGCCACCGGTA was introduced generating a pDM628 vector without the RRE fragment (pDM628ΔRRE). The RRE fragments derived from the patients were PCR-amplified using the XmaI-RREF (TCTTCAGACCCGGGGGAGGAGATA) and SpeI-RRER (GTTGTATATTAAACTAGTGTAATTCTCAA) primers from the same full-length Env-expressing plasmids that were used to generate the RRE-expressing plasmids for the *in vitro* RNA production. Subsequently, both the pDM628ΔRRE vector and the RRE fragments derived from the patient samples were digested with the restriction enzymes *SpeI* and *XmaI*, purified and ligated together to generate pDM628 plasmids with the patient-derived RRE (pDM628-RRE-expressing plasmids). All plasmids were sequenced to verify that no changes were introduced during their construction.

### Rev-dependent RNA transport assay

Twenty-four hours before transfection, 5×10^5^ 293T cells/well were seeded into 24-well tissue culture plates in 0.5 mL of cell culture medium. The following day, each well was co-transfected with pCMV-Rev (200 ng/well) and pDM628-RRE-expressing plasmids (800 ng/well) using the Calphos Mammalian transfection kit (Clontech Laboratories). In parallel, to determine the background levels, all the pDM628-RRE-expressing plasmids (800 ng/well) were also co-transfected with pcDNA 3.0 (200 ng/well). In addition, as a control, cells were also co-transfected with pDM628ΔRRE or pDM628 (800 ng/well each, negative or positive control, respectively) with pCMV-Rev and pcDNA 3.0 (200 ng/well). Cells were collected 24 h post-transfection and luminescence was measured by using the luciferase assay reagent (Britelite Luminiscence Reporter Gene Assay System, Perkin Elmer Life Sciences) with a Luminoskan Ascent luminometer (Labsystems, Spain). The fold change was calculated by the normalization of the relative light units to the pDM628 signal, and relative to the reporter without Rev. In dose-response assays, 293T cells were co-transfected with a constant amount of pDM628-RRE-expressing plasmids (800 ng/well) and three decreasing concentrations of pCMV-Rev (200, 20 and 2 ng/well) and luminescence was detected as described above. Results were obtained through three independent transfections performed in triplicate and data was presented as the mean +/− SEM, using Graph Prism 5.0 software (GraphPad Software, La Jolla, CA, USA).

### RRE-recombinant viruses

A p83.10ΔRRE vector was constructed using the same PCR strategy as used in the pDM628ΔRRE construction. The RRE deletion (corresponding to nucleotides 7647–8128) was created using the primers and PCRs described above using the p83.10 plasmid. RRE fragments from patients were amplified from full-length Env-expressing plasmids and cloned into the p83.10ΔRRE vector as described above to generate p83.10-RRE-recombinant plasmids. Production of replication competent RRE-recombinant viruses was performed by the co-electroporation of 4×10^6^ MT-4 cells with p83.2 and p83.10-RRE-recombinant plasmids (6 µg each) linearized with *EcoRI*. After 7 days, culture was diluted at a density of 0.2×10^6^ cell/mL and after 3–4 days the cell-associated RNA was isolated. As controls, co–electroporation of p83.2 parental+p83.10 and p83.2+p83.10ΔRRE were included in all the experiments. Viral p24 production in cell-free supernatants was measured by an enzyme-linked immunosorbent assay (ELISA) (Innogenetics).

### HIV-1 RNA isolation and qRT-PCR

Nuclear and cytoplasmic RNA fractions were isolated as described previously [33]. Approximately 1×10^6^ cells were washed in cold PBS and plasma membranes were lysed on ice for 10 min in cold lysis buffer containing 10 mM TrisCl [pH 7.5], 140 mM NaCl, 1.5 mM MgCl_2_, 0.5% (vol/vol) Igepal, 1,000 U/ml RNase inhibitor, and 1 mM dithiothreitol. After the lysis, a short (5-min) low-speed centrifugation at 350×*g* and 4°C was carried out, and the cytoplasmic supernatants were transferred to new tubes where an additional high-speed centrifugation step (5 min at 4°C and 13,000×*g*) was performed. The supernatant was collected as the cytoplasmic fraction. Nuclear pellet was washed in the same cold lysis buffer, centrifuged at 350×*g* for 2 min at 4°C and resuspended in RNeasy lysis buffer (RNeasy plus Mini Kit, Qiagen) for 5 min on ice. After centrifugation through a QIAshredder column and a gDNA Eliminator column (Qiagen) the flow-through was collected as the nuclear fraction. Subsequently, RNA was isolated from both cytoplasmic and nuclear fractions using the RNeasy kit adding a DNase-treatment on column (RNase-Free DNase Set, Qiagen). In all RT-PCR experiments control reactions were included lacking the RT enzyme or template. All such controls were negative. Each extracted RNA was analyzed in triplicate by qRT-PCR using the Power SYBR® Green RNA-to-CT™ 1-Step Kit (Life technology) according to the manufacturer's instructions. Primers were as follows: unspliced transcripts forward: GTCTCTCTGGTTAGACCAG; unspliced transcripts reverse: CTAGTCAAAATTTTTGGCGTACTC [34]; pre-GAPDH forward: CCACCAACTGCTTAGCACC; pre-GAPDH reverse: CTCCCCACCTTGAAAGGAAAT [35]; GAPDH forward: TCTCCTCTGACTTCAACAGCGAC; GAPDH reverse: CCCTGTTGCTGTAGCCAAATTC. QRT-PCR cycling program: 50°C for 10 min and 95°C for 5 min, followed by 40 cycles at 95°C for 10 s and 60°C for 30 s. The specificity of the PCR product was examined by a dissociation curve, and data were analyzed using the 2^(−ΔΔCt)^ relative quantification method [36]. GAPDH was used for normalization and the RREWT (40Q-45L) was used as the calibrator control. ΔΔCt = (C_UNSPLI_−CT_GAPDH_)_mutants_−(Ct_UNSPLI_−CT_GAPDH_)_WT_. Data (mean +/− SEM) were derived from three independent experiments, with triplicate samples for each PCR and analyzed with Graph Prism 5.0 software (GraphPad Software, La Jolla, CA, USA).

### HIV-1 DNA isolation and qPCR

From the same amount of cells used for RNA isolation, DNA was isolated using QIAamp DNA Blood kit (Qiagen, Spain) according to the manufacturer's instructions. Quantitative real-time PCR was performed in triplicates using a TaqMan Universal Master Mix (Applied Biosystems, Spain) and the following primers and probes: DNA proviral-688F: GACGCAGGACTCGGCTTG; DNA proviral-809R: ACTGACGCTCTCGCACCC; DNA proviral-735T: FAM- TTTGGCGTACTCACCAGTCGCCG- TAMRA; CCR5-576F: TCATTACACCTGCAGCTCTCATTT; CCR5-726R: ACACCGAAGCAGAGTTTTTAGGAT; CCR5-661T: VIC-CTGGTCCTGCCGCTGCTTGTCA-TAMRA to detect total HIV DNA and CCR5, respectively. All real-time PCR reactions were performed in triplicates and using ABI Prism 7000 (Applied Biosystems, Spain). CCR5 was used as an endogenous control to normalize for variations in the amount of starting material in each PCR reaction and the RREWT (40Q-45L) was used as the calibrator control. Data were analyzed using the 2^(-ΔΔCt)^ method as explained above.

## Results

### Sequence variation of the RRE and Rev derived from primary isolates under ENF pressure

**Figure 1:**
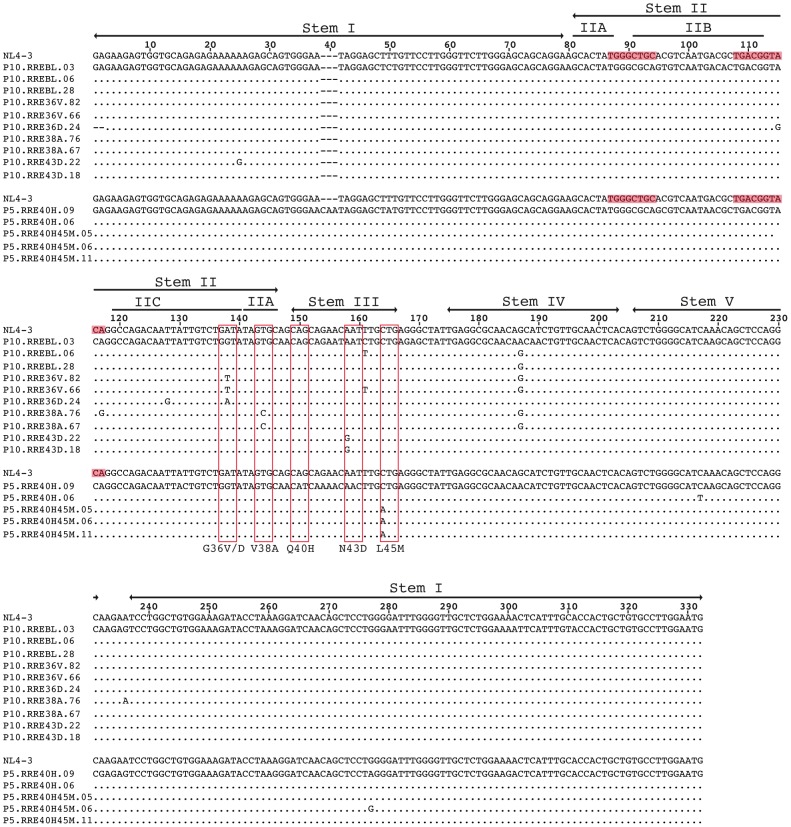
Alignment of patient-derived RRE variants used for functional analysis. Multiple nucleotide alignment of the full-length patient-derived RRE variants used for prediction of secondary structures and functional analyses. Several full-length RREs derived from two patients (patient 10: P10; and patient 5: P5) were amplified from plasma samples, cloned and sequenced. Boxes highlight the nucleotides coding ENF resistance mutations G36V/D, V38A and N43D for patient 10; and Q40H and L45M for patient 5. The characteristic five stem-loop regions of the RRE are identified on top of the sequence. Variants were designated according to the patient number, amino acid change in gp41 and clone number. The shaded areas indicate the reported high-affinity binding site of Rev located in the stem II. The NL4-3 RRE sequence is included for comparison.

In a previous report, we characterized gp41 proteins derived from 13 heavily pre-treated HIV-1-infected patients receiving an ENF-containing salvage therapy [25]. Several drug resistance-associated mutations were detected along the entire gp41 ectodomain, mainly mapping in the HR1/RRE region. Two patients with mutations at positions 36, 38 and 43 (patient 10, P10) and at positions 40 and 45 (patient 5, P5) were selected in order to analyze the main gp41/RRE changes selected *in vivo* under ENF-treatment. Plasma samples were obtained from these two patients at different time points, and used to construct full-length Env-expressing plasmids. Following sequencing, 10 plasmids were selected from patient 10, and 5 plasmids from patient 5. Each clone was identified according to the amino acid change in gp41 that it displayed (Figure 1). As expected, the RRE region was highly conserved and the nucleotide changes found were coding for the resistance mutations selected by the ENF treatment (HR1 in gp41 which co-inside in the stems IIC, IIA and III in the RRE), with other minor changes throughout the sequence (Figure 1). Only two variants presented substitutions in the high-affinity site for Rev in the stem-loop IIB (P10.RRE36D.24 and P10.RRE38A.76). All selected RRE sequences were PCR-amplified and cloned into an appropriate expression vector for activity assays.

In addition, it has been suggested that changes in the RRE that emerged after ENF treatment can be compensated by changes in the Rev protein [37]. More recently, a functional analysis of Rev-RRE paired samples have shown a correlation between Rev amino acid and RRE nucleotide sequences may co-evolve, *in vivo* [22]. Thus, in order to determine if any of our RRE variants were associated with specific changes in the Rev protein, the first two exons of Rev were amplified from a few of the full-length Env-expressing plasmids. For patient 10, no changes were observed in the known functional domains of the protein, whereas, samples with the double mutation (RRE40-45) from patient 5 had amino acid changes in the first oligomerization domain (Figure S1).

### Prediction of patient-derived RRE secondary structures

**Figure 2:**
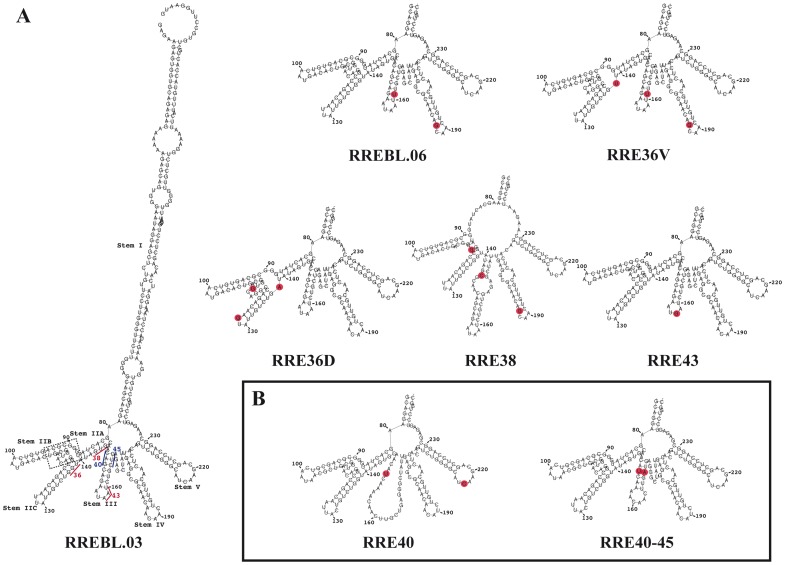
Predicted secondary structures of patient-derived RRE variants. The prediction of the secondary structure of our RRE sequences was generated including the 329-nucleotide sequence of the patient-derived RREs in the RNA Fold Web server. The five well-defined stem-loop structures, including the branched stem-loop IIB that is critical for the binding of the Rev protein, were identified for most of the sequences. A) Predicted secondary structure of RRE from patient 10. Predicted secondary structure of the complete RRE of a BL clone from patient 10 is shown. The nucleotides encoding the amino acid changes associated with ENF resistance are underlined and a dotted box encloses the high-affinity Rev binding site. Stems loops II-III-IV and V for representative samples for each substitution present in Patent 10 are shown. Nucleotide changes present in the RRE variants with regard to the BL clone are marked with filled red circles. B) Representative RRE structures obtained from samples of patient 5.

For patient 10 three individual baseline (BL) clones without ENF-resistance mutations (RREBL03, 06 and 28) were obtained with a nearly identical nucleotide sequence. Only two nucleotide changes were present at positions 161 and 187 in the Stems III and IV, respectively (Figure 1). When the BL RRE sequences were subjected to prediction of RNA folding using the Vienna RNA Fold program [29] the previously described characteristic 5 stem-loop subdomains were observed [6], [7]. Minor variations (highlighted in red in RREBL.06 in Figure 2A) led to the lengthening of the stem IV by the formation of an additional base pair at the end of the stem. The G36V and G36D mutations in gp41, corresponding to IIC of the RRE, derive from the nucleotide substitutions (in boldface) G**G**U to G**U**U and G**A**U, respectively (highlighted in red in Figure 2A). This nucleotide change resulted in the disruption of the second base pair of the stem IIC. This may cause instability of the terminal base pair and formation of a larger bulge in between stem IIA IIB and IIC. Although this structural variation was minimal, it could potentially disturb the affinity-binding site of Rev, and result in a functional impairment. The other nucleotide changes present at positions 115 and 128 in the RRE36D (Figure 1) had no impact on the predicted structure. In contrast, significant changes were observed in the predicted secondary structures of the RRE38A variants, confirming previous structural data [38]. The single nucleotide substitution G**U**G to G**C**G, resulted in a six-way rather than a five-way central RNA junction in the predicted structure, causing a complete abrogation of stem IIA and the Rev-binding site, creating a larger bulge at the end of the stem I and a stem-bulge structure before the stem III. The nucleotides, coding the amino acid at position 43 in gp41, are located in a single-strand bulge region of the stem III and changes in these positions (**A**AU to **G**AU) did not produce any alteration in the predicted RRE structure. For patient 5, baseline plasma samples were not available, and only plasmids with the Q40H change or with the double mutation Q40H-L45M in gp41 were obtained (Figure 1). The nucleotides coding for amino acids 40 and 45 are located complementary to each other in the stem III (underlined in blue in Figure 2A in a BL clone from patient 10). The emergence of Q40H (RRE40) completely abrogated the predicted structure of the stem III, which was restored in the variants with the double mutation Q40H and L45M (RRE40-45) (Figure 2B)[37].

### *In vitro* RRE-Rev binding assay

**Figure 3:**
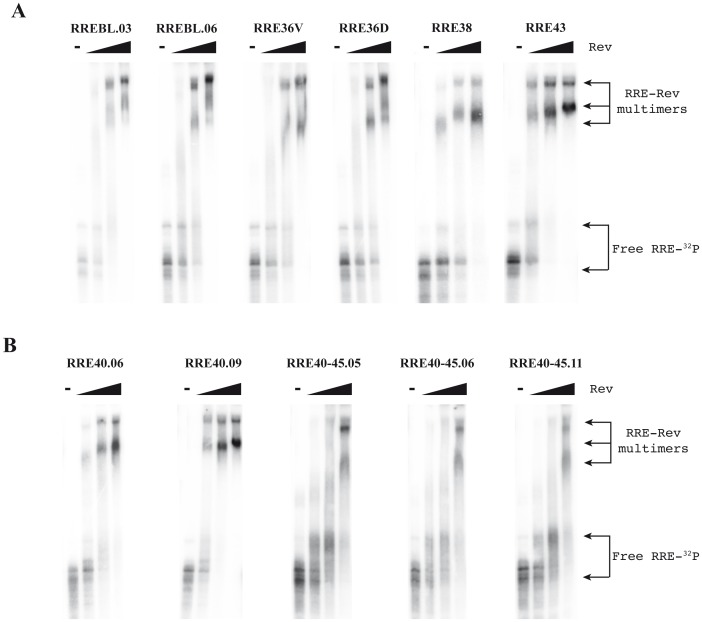
Binding and multimerization of selected RRE variants using electro-mobility shift assay (EMSA). ^32^P-labeled RRE RNA was synthesized *in vitro* and a constant amount was incubated in a binding reaction with increasing amounts of Rev protein (20 ng, 40 ng and 80 ng) or without Rev The resulting complexes were analyzed by electrophoresis in a native polyacrylamide gel. The gel was dried and examined using a phosphorimager. Binding pattern observed after incubation of RRE variants is shown in A) from patient 10, and B) from patient 5.

The functional capacity of the RRE variants was first accessed *in vitro* by measuring Rev binding ability in an electrophoretic mobility shift assay (EMSA). A constant amount of radiolabeled *in vitro*-transcribed RRE RNA was incubated with increasing amounts of the Rev protein and the resulting complexes were separated in a polyacrylamide gel and detected by phosporimaging (Figure 3). Efficient binding of Rev to the RREBL clones, measured by the formation of slower migrating complexes, was observed in all concentrations of Rev used. Regardless of the sequence or the extent of variation in the predicted secondary structure, the RRE variants isolated from patient 10 (RRE36D, RRE36V, RRE38 and RRE43) bound Rev protein with similar efficiency as RREBL (Figure 3A), suggesting that despite the prediction when RRE variants are synthesized *in vitro*, they form similar structures which was further supported by the similar migration observed with the RRE alone. The RRE40 variant from patient 5 formed complexes similar to the RREBL from patient 10. Surprisingly, a change in the Rev-RRE complex formation pattern was observed in all RRE40-45 clones tested (Figure 3B). The double mutants failed to form multimers, which were present only when the highest concentration of Rev was used.

### Secondary structure prediction and *in vitro* Rev-RRE binding in variants with changes at positions 40 and 45

**Figure 4:**
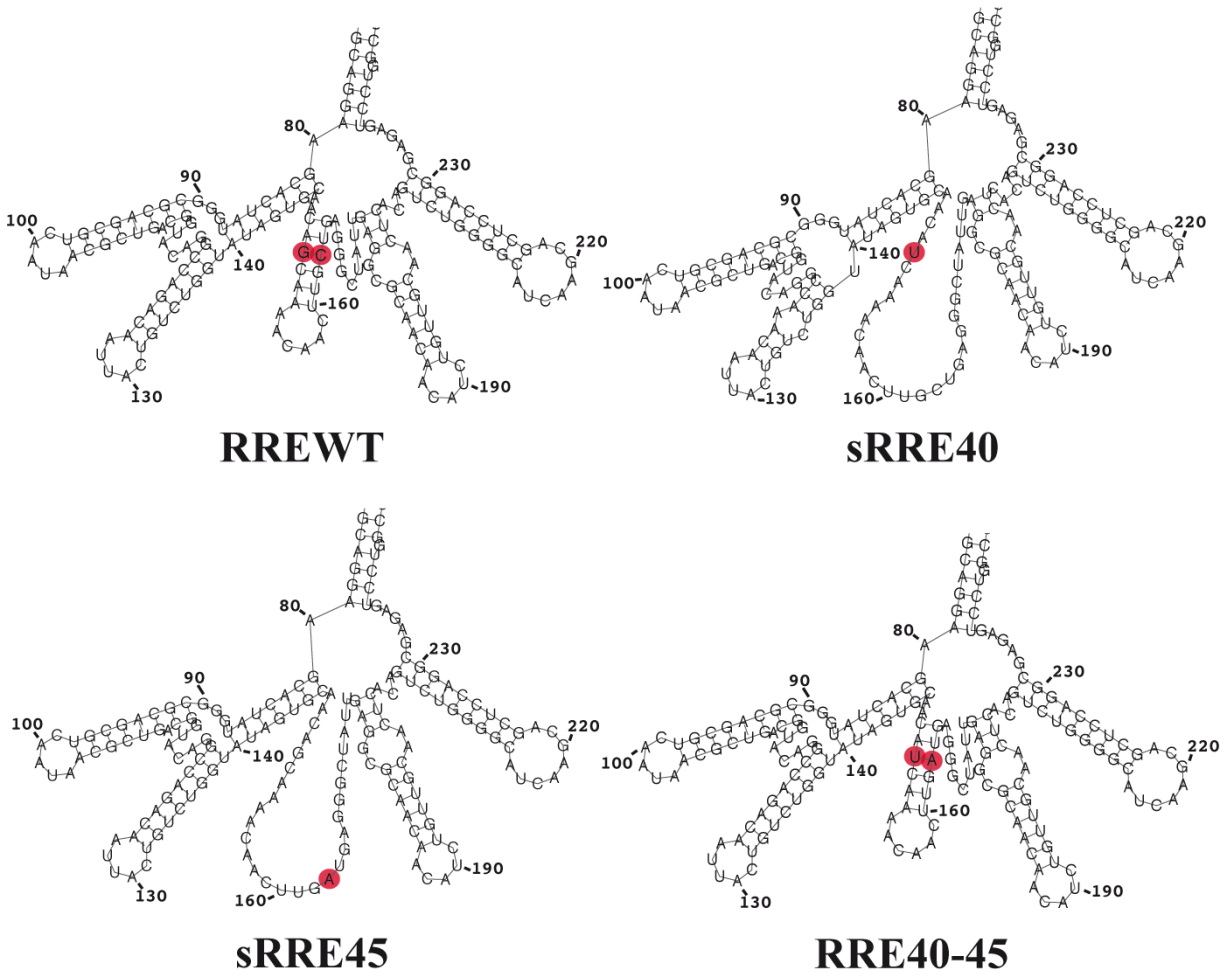
Predicted secondary structures of RRE variants constructed by site-directed mutagenesis. RRE variants with nucleotide substitutions at position 40 sRRE40 (Q40H), at position 45 sRRE45 (L45M) or without any, WT (40Q-45L), were generated by site-directed mutagenesis using as a template a RRE40-45 clone (Q40H-L45M). The sequences of these constructed clones were subjected to RNA fold analyses as described in Figure 2. Nucleotides that were substituted are highlighted in the filled red circles.

**Figure 5:**
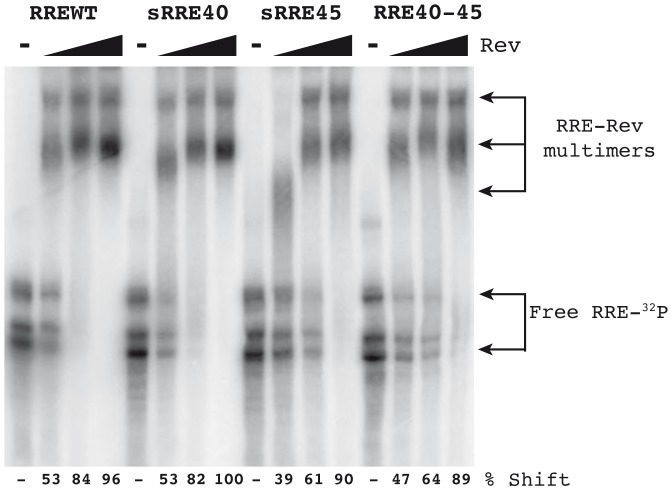
*In vitro* Rev-RRE binding assay. Electro-mobility shift assay (EMSA) in the absence or in the presence of 20 ng, 40 ng or 80 ng of Rev protein with the different RRE RNA variants (RREWT, sRRE40, sRRE45 and RRE40-45). The reaction products were separated in a polyacrylamide gel and the quantification of the gel shifts are displayed.

For this patient, baseline samples were not available and plasmids with the change L45M were not rescued. In order to further characterize changes at positions 40 and 45, single sRRE40, sRRE45 and RREWT (40Q-45L) sequences were generated by site-directed mutagenesis using the RRE40-45 double mutant as a template (see sequences in Figure S2). The RREWT (named WT in order to distinguish it from the RREBL isolated from patient 10) was generated by changing the nucleotides 151 and 164 (Figure S2), resulting in the reversion of the resistance mutations 40 and 45. The predicted secondary structure of both single mutants were similar, displaying a previously reported disrupted stem III [37] (Figure 4). A minor difference, the formation of a stem-bulge-stem structure on the site-directed sRRE40 mutant at the stem IIC, due to a nucleotide change (G to A) at position 123 (Figure S2 and Figure 4), was observed. The constructed RREWT revealed an identical structure to RRE40-45 (Figure 2B and Figure 4)[37]. In the *in vitro* Rev-RRE binding assay, the percentage of shift from the gel was calculated as a fraction of RNA bound (total bound RNA in all bands divided by the sum of bound and unbound RNAs) (Figure 5). The formation of complexes between the RREWT and Rev was quantified, showing 53%, 84% and 96% of shift at 20 ng, 40 ng, and 80 ng of Rev, respectively. Compared to the values obtained with the RREWT, and as we previously observed, there was a decrease in the RRE40-45-Rev binding capacity (64% of shift with 40 ng of Rev). Higher levels of Rev were necessary to form multimers. Although even at the highest concentration of Rev tested, the percentage of shift was slightly lower for the double mutant than in the RREWT variant (89% with 80 ng of Rev). The new single mutants, displayed a different pattern of migration in the presence of Rev. The sRRE40 behaved as the RREWT while sRRE45 showed the same impairment observed with the RRE40-45. However, in the *in vitro* system, each mutant appeared to adopt a similar structure with an equal migration in the absence of Rev. Thus, the sole nucleotide change at position 164 (amino acid 45) changed the efficiency with which the RRE bound Rev. This finding indicated that the RRE-Rev binding capacity in this *in vitro* assay could be determined by the primary sequence present by the variants.

### Rev-dependent RNA transport assay of RRE variants in a reporter assay

**Figure 6:**
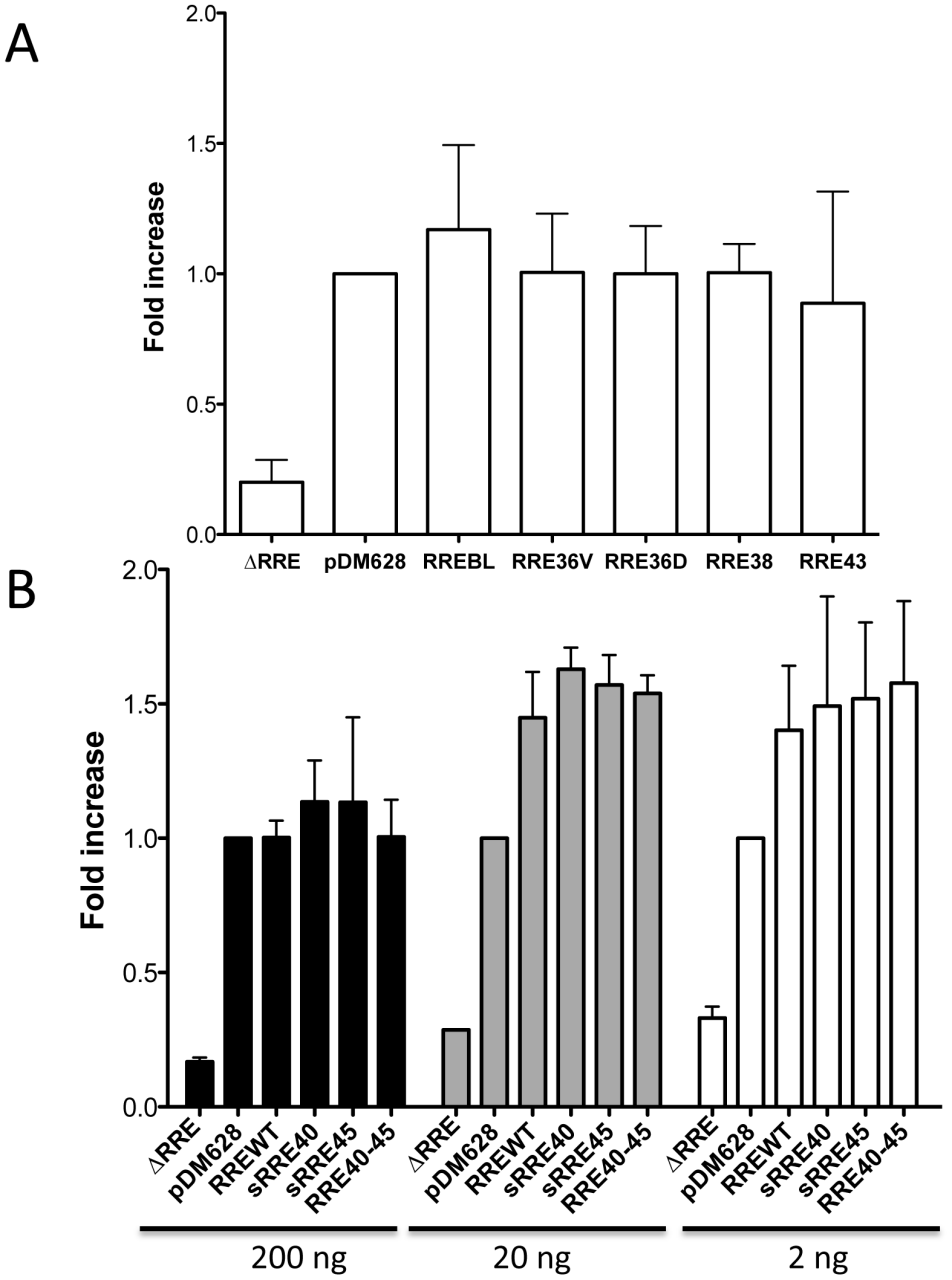
Rev-dependent RNA transport. 293T cells were co-transfected with the constructed RRE variants, pDM628 or pDM628ΔRRE with or without pCMV-Rev. The export levels of the different variants were quantified by luminescence and corrected by the background signal from the luminometer noise or due to Rev-independent transport. This correction was performed to each sample by subtracting the luminescence that was measured when the cells were transfected without Rev (replaced with pcDNA 3.0). And finally, corrected luminescence values were calculated as the fold-change increase, which was performed by dividing the corrected luminescence of each plasmid by the corrected luminescence of the pDM628 plasmid. A) Rev-RRE mediated export from RNA variants containing changes at positions 36, 38 and 43, evaluated in the presence of Rev (ratio 1:5, Rev:RRE). B) Cytoplasmic export of RRE variants with changes at positions 40 and 45. Rev-dependent transport of the pDM628-based RRE variants: WT (40Q-45L), sRRE40 (Q40H), sRRE45 (L45M) and the double mutant RRE40-45 (Q40H-L45M); in the presence of three different concentrations of pCMV-Rev (200 ng, 20 ng or 2 ng per well). Data represent the mean +/− SEM of 3 independent experiments performed in triplicate transfections.

The data obtained in the *in vitro* Rev-RRE binding assay suggested that most of the RRE variants bind to the Rev protein with efficiency similar to RREBL. This is in accordance with the Rev-RRE interaction being essential for viral replication. To further investigate the functionality of our patient-derived RREs, a Rev-dependent RNA transport assay in a mammalian cell was performed. In order to examine the capacity of the RRE to recruit Rev protein and facilitate the viral RNA export the cytoplasm we employed a transient co-transfection assay. The assay included a Rev-expression plasmid and a Rev-dependent, luciferase-based reporter plasmid (pDM628) harboring a single intron that included both the RRE and the luciferase coding sequence, which only is expressed from the unspliced RNA [31]. The RRE was replaced with each of the RRE variant by cloning them into a constructed reporter plasmid lacking the RRE (pDM628ΔRRE). The constructs were co-transfected into 293T cells with a Rev-expressing plasmid. The pDM628 and the Rev-expression plasmids were titrated to determine the best ratio that induced the maximum luminescence. RNA export in these cells is dependent on the RRE region, due to a lack of RNA export when using the pDM628ΔRRE plasmid (Figure 6). The luciferase production in 293T cells was stimulated three-four-fold by Rev, when transfected with pDM628 (data not shown) [31]. The export level of the different variants was calculated as the fold-change increase compared with the pDM628 vector, once the luminescence obtained without Rev was subtracted. Export level analysis indicated that Rev-dependent transport of RRE36V, RRE36D, RRE38 and RRE43 was similar to that of RREBL or the pDM628 plasmid (Figure 6A). For site directed RRE variants, with changes at positions 40 and 45, a dose-response RNA transport assay was performed. Since sRRE45 and RRE40-45 showed a differential behavior in the *in vitro* RRE-Rev binding assay when different concentrations of Rev were used, it is possible that differences in RRE function would be dependent on the amount of Rev activity present in the cell. No changes were observed in the level of Rev-dependent RNA transport for any of the variants compared to pDM628 when 200 ng of Rev was used (Figure 6B). However, when the amount of RRE was kept constant (800 ng) and the amount of Rev was reduced to 20 ng or 2 ng, all our RREs variants yielded a higher signal than pDM628. Nevertheless, when comparing the RRE constructed variants to each other, the activities were similar to the RREWT. This is in contrast to the binding differences observed *in vitro*.

### Rev-dependent RNA transport in RRE mutant viruses

**Figure 7:**
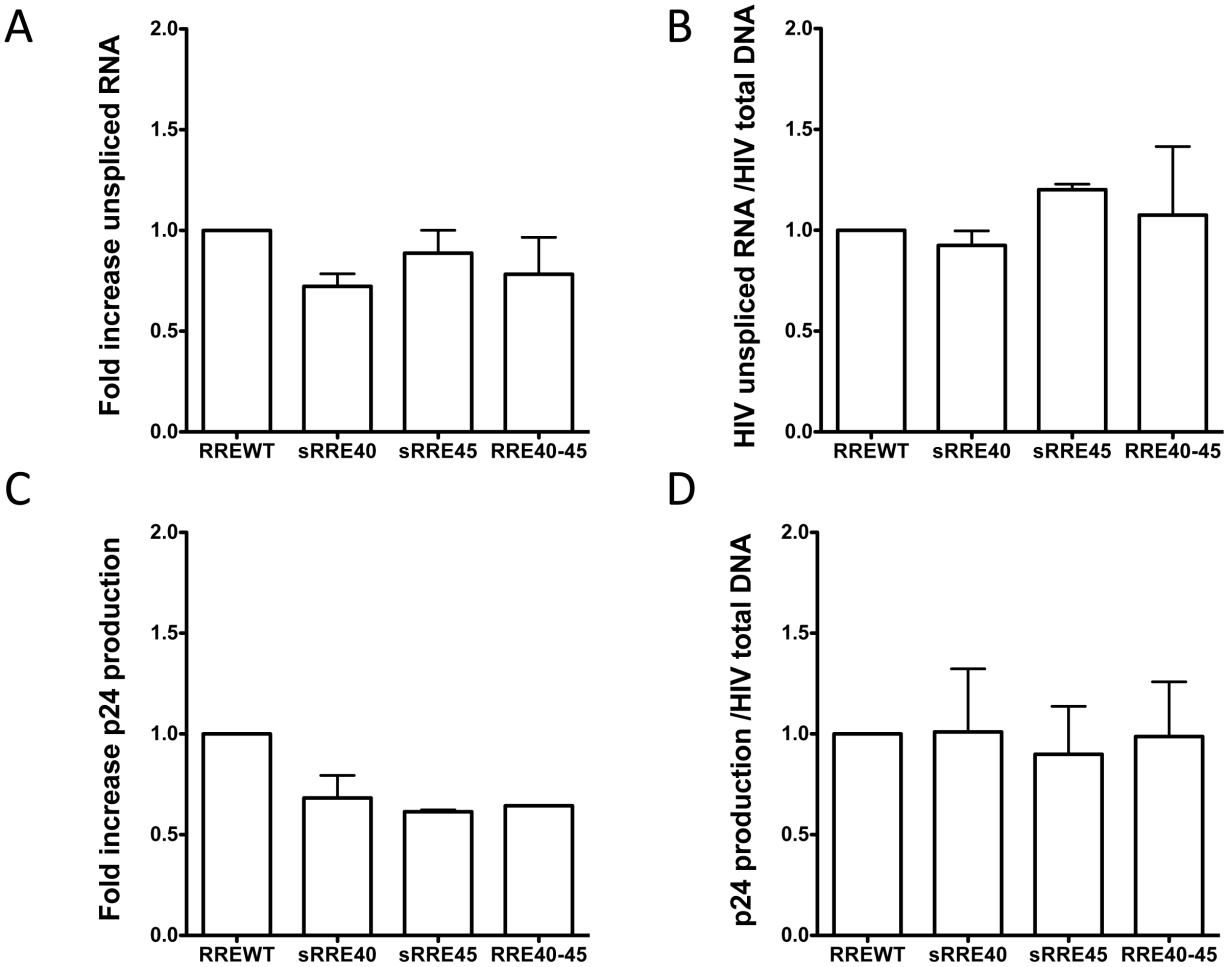
Functional analyses of RRE with changes at positions 40 and 45 in lymphoid cells. The lymphoid cell line MT-4 was co-electroporated with the NL4-3 hemigenomic plasmids p83-2 and with the p83-10 constructed variants, which only differed in their RRE region. A) Fold increase of cytosolic unspliced RNA in infected cells. Levels of unspliced RNA in the cytoplasmic fractions were determined by qRT-PCR. The fold change was calculated by the relative quantitation method 2(^−ΔΔCt^). GAPDH was used for normalization and RREWT (40Q-45L) as a calibrator. Data (mean +/− SEM) is derived from three independent experiments, with triplicate samples in each PCR. B) Quantification of the transcriptional activity per infected cell. The ratio between the levels of unspliced HIV RNA and total HIV DNA (2(^−ΔΔCt^) Unspliced HIV-1 RNA/2(^−ΔΔCt^) Total HIV-1 DNA) was calculated. Total HIV DNA content was determined in cells by qPCR using the same approach as described with the unspliced RNA levels. Data (mean +/− SEM) is derived from three independent experiments, with triplicate samples in each PCR. C) P24 protein production in cell-free supernatants. The p24 present in the supernatant of the cultures was quantified by ELISA from the same time-point that the RNA and DNA levels were determined. Data (mean +/− SEM) is derived from two independent experiments. D) Normalized p24 protein production. Raw p24 values were normalized to copies of total HIV-1 DNA to correct for differences in electroporation efficiency and gp41 function. Data (mean +/− SEM) is derived from two independent experiments.

The Rev-RRE functionality was evaluated using recombinant viruses in lymphoid cells measuring the amount of unspliced RNA levels present in the cytoplasm. RREWT (40Q-45L), sRRE40, sRRE45 and RRE40-45 fragments were cloned into a constructed NL4-3 hemigenomic p83.10ΔRRE. To avoid the excess of RNA and DNA present in the cytoplasm after electroporation, cells were extensively diluted 7 days post-electroporation. After 10–11 days of culture nuclear and cytoplasmic RNA was obtained and RT-PCR using specific primers for unspliced GAPDH RNA (pre-GAPDH) was performed to confirm the purity of the fractionation procedure [35]. The unspliced GAPDH pre-mRNA was found only in the nuclear fractions, indicating that the leakage of nuclear unspliced mRNAs into the cytoplasm was minimal (Figure S3). To measure the amount of full-length (unspliced) HIV-1 RNA in the cytoplasm a quantitative RT-PCR was performed with primers targeting the LTR region, and the fold change was calculated by the 2^(−ΔΔCt)^ method. GAPDH was used to normalize the values and the RREWT was used as the calibrator. To confirm the dependency on RRE for accumulation of unspliced RNA in the cytoplasm, a p83.2+p83.10ΔRRE control was included in all the experiments. The levels of unspliced RNAs in the cytoplasm were undetectable with the deletion of the RRE, showing that in our cellular model the nuclear export of unspliced RNA was totally Rev-RRE dependent. The amounts of unspliced RNA detected in the cytoplasm of cells electroporated with the plasmids containing the sRRE40, sRRE45 and RRE40-45 were slightly lower compared to the WT (mean of 0.72, 0.88 and 0.77, respectively). This suggests, a small defect in the transport of the unspliced HIV-1 RNA from the nucleus to the cytoplasm (Figure 7A). However, the quantification of the HIV RNA copies need to be normalized by the levels of HIV DNA present in the culture, which may vary due to differences in the electroporation efficiency and/or changes in the function of gp41. Total HIV DNA content was determined by qPCR and the fold change was also calculated by the 2^(−ΔΔCt)^ method. CCR5 was used to normalize the values and the RREWT was used as the calibrator. Upon normalization, the activity per infected cell (ratio between the levels of unspliced HIV RNA and total HIV DNA) [39], [40] of the different RRE variants was obtained. All RRE variants displayed the same function and similar levels of normalized unspliced RNA in the cytoplasm than RREWT (mean of 0.92, 1.2 and 1.07 for the sRRE40, sRRE45 and RRE40-45, respectively. Figure 7B).

To evaluate whether our mutants had alterations in functions other than RNA transport, differences in virus production were also evaluated. Therefore, we measured the amount of p24 protein in the culture supernatant at the same time point in which the RNA was determined. Similar to the results obtained with the levels of unspliced RNA, a lower p24 production with the RRE variants in comparison with the RREWT (Figure 7C) was observed. However, the amount of p24 produced per infected cell was the same for all RRE variants (Figure 7D) when the p24 values were normalized to DNA levels. Taken together, these results demonstrated that Rev-RRE function is largely maintained in *in vivo*-selected RRE variants, indicating an extensive conformational plasticity of the RRE structure.

## Discussion

The HIV-1 Rev-RRE pathway has been shown to be critical for viral gene expression and replication, thereby implicating it as a potential clinical target. However, it is unclear whether the interaction and function in primary isolates are dependent on sequence, secondary structure, or the overall three-dimensional structure of the element. Therefore, it is important to understand how changes in these components are affected by selective pressure in the Rev-RRE activity.

In the present study, some RRE variants were obtained from HIV-1 infected patients who had virological failure to an ENF-containing salvage therapy, and from which we had previously identified the presence of changes in the RRE/gp41 region [25]. We investigated the impact of substitutions introduced *in vivo* after developing resistance to ENF on secondary structure and function of RRE.

The structure of the RRE has been investigated by a number of different techniques [2], [6], [7], [12], [41]–[43] including computational prediction methods. Our RRE variants were subjected to computational prediction of the secondary structure using Vienna RNA fold (58). We observed that although most of the variants retained the essential stem-loop formation with the five stem-loops emerging from a central bubble [2], [6], [18], [42], some had drastic structural changes compared to the RREBLs, as seen previously in studies analyzing ENF-selected variants [37], [38], [44]. The functional impact of these changes was determined by studying different steps in the Rev-RRE pathway. We first analyzed the *in vitro* binding between Rev and RRE assessed by an electrophoretic mobility shift assay (EMSA). All the variants displayed a similar migration in the absence of Rev, which does not support the structural changes displayed with the prediction. In addition, differences in the ability of Rev to bind to the different variants were observed only for the RRE45 and RRE40-45. Thus, the impairment was surprisingly not associated with the alteration in the predicted structure as suggested, but with a nucleotide change in the sequence. This mutated nucleotide, located at position 164 in both RRE45 and RRE40-45 variants, lies outside the primary Rev binding domain, highlighting the importance of other regions in the Rev-RRE binding. These findings are consistent with previous results in which laboratory-adapted clones or primary-isolated sequences were used to show that limited changes throughout the RRE could alter the Rev binding [14], [15], [22]. Although there is a general correlation between *in vitro* Rev-RRE binding, and RRE function, binding and function do not always go hand in hand. As previously shown, functionally inactive RRE variants were able to bind Rev, even more avidly than RREWTs [8]. Thus, we assessed the ability of the Rev protein to facilitate nuclear export of the unspliced mRNA to the cytoplasm using a reporter assay. The ability to direct transport of intron containing mRNA into the cytoplasm was similar to the plasmid control (pDM628). In fact, transport increased as the amount of Rev was reduced, further proving previously published material, in which RRE isolated from patients were significantly more active than the NL4-3 RRE when paired with NL4-3 Rev [22]. The differences in Rev-binding measured in the EMSA experiments and the non-correlation with the data obtained in the Rev-dependent transport assay could be explained by the fact that in this system the transient transfection of 293T cells results in a over-expression of Rev protein [35], even when the Rev plasmid was decreased 100 fold, preventing the detection of small changes in Rev-RRE binding. Therefore, we studied the activity of different RRE variants under a more natural expression system using infected lymphoid cells, simulating an infected patient. After normalization and supporting the data obtained with the transient assay, no differences in the transcriptional activity per infected cell or in p24 production were observed among variants, further suggesting that all the RREs were functional and likely structurally equivalent.

Finally, it has been revealed that specific Rev mutations are associated with ENF treatment and with some specific changes in gp41, suggesting that changes in the RRE that appeared after ENF treatment could be compensated by mutations in the Rev protein [37]. Recently, a functional analysis of Rev-RRE paired samples has revealed that *in vivo* there is a relationship between the sequence variation of Rev and RRE [22]. In our samples, some changes in the Rev protein were observed, but none were associated with ENF treatment. Most of the changes were detected in samples with the double mutation 40–45, in which various changes could be detected in the first oligomerization domain (OD1). Thereby suggesting cooperative mutations in the entire Rev-RRE pathway may have co-evolved. A functional analysis of paired Rev-RRE sequences was not performed, and therefore we cannot exclude the possibility that these changes in Rev could impact the Rev-RRE activity. Nevertheless, based on a previous study in patients, the RRE, and not Rev, appears to be the driver of the overall changes in activity [22].

Taken together, these results demonstrate that Rev-RRE function is maintained in RRE variants selected *in vivo*, indicating an extensive conformational plasticity of the RRE structure.

In conclusion, the RRE is a dynamic structure capable of structural rearrangements that *in vivo* could assume alternative structures that are functionally equivalent. Therefore, changes in the nucleotide sequence or in the predicted secondary structure might not always reflect the real impact on the function of the RRE.

The malleability of the RRE under selective pressure is still unknown. Our study provides new insights into the *in vivo* structural plasticity of the RRE, showing that under pressure the virus is able to overcome changes in RRE without displaying a functional impairment. Therefore, these results should be taken into account in the development of antiviral agents that target the Rev-RRE complex.

## Supporting Information

Figure S1 **Amino acid alignment of patient Rev variants.** Sequences from the Rev protein (Exon 1 and 2) were obtained from some of the envelope-expressing plasmid used for RRE analysis. The shading indicates the described functional domains of the protein. OD1, first oligomerization domain; ARM, arginine-rich motif; OD2, second oligomerization domain; NES, nuclear export signal.
(TIF)

Figure S2 **Multiple nucleotide alignment of RRE variants with nucleotide substitutions coding amino acids 40 and 45.** The sRRE40 (Q40H), sRRE45 (L45M), and RREWT (40Q-45L) variants were created from a double mutant variant RRE40-45 (Q40H-L45M) by site-directed mutagenesis, cloned into a pPCR-Script vector and sequenced. Boxes highlight the nucleotides encoding the amino acids changed.
(TIF)

Figure S3 **Real-Time RT-PCR of pre-GAPDH and GAPDH from nuclear and cytoplasmic fractions.** Nuclear and cytoplasmic RNA fractions were isolated under standard conditions and were used to amplify the unspliced GAPDH (pre-GAPDH) and the total GAPDH with specific primers.
(TIF)
