# Supplementary figures and images for: Functional Analyses Reveal Extensive RRE Plasticity in Primary HIV-1 Sequences Selected under Selective Pressure
Source: PLoS One. 2014 Aug 29;9(8):e106299. doi: 10.1371/journal.pone.0106299 (PMC4149556; doi:10.1371/journal.pone.0106299)

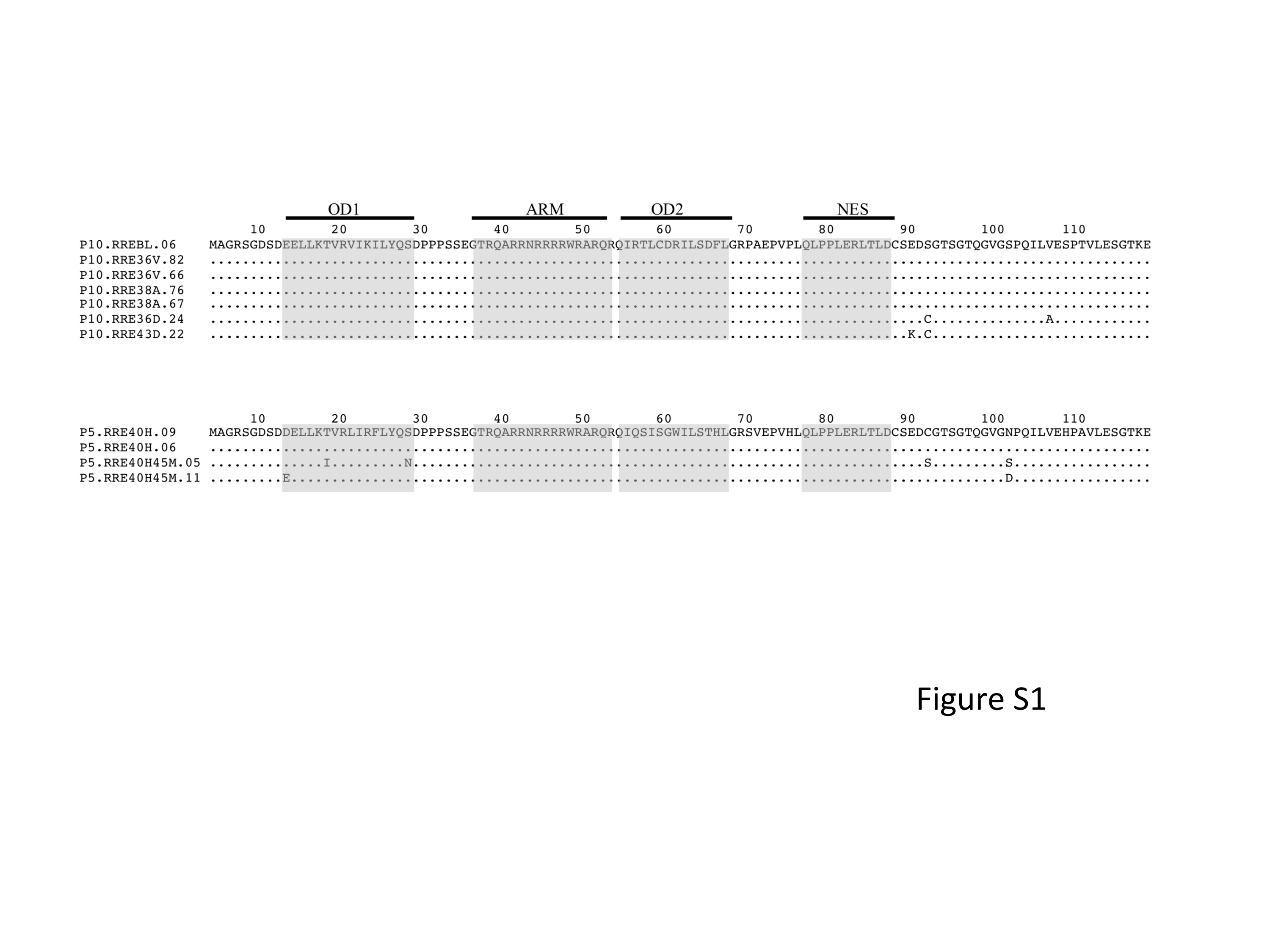

Supplement: Figure S1 — Amino acid alignment of patient Rev variants. Sequences from the Rev protein (Exon 1 and 2) were obtained from some of the envelope-expressing plasmid used for RRE analysis. The shading indicates the described functional domains of the protein. OD1, first oligomerization domain; ARM, arginine-rich motif; OD2, second oligomerization domain; NES, nuclear export signal. (TIF) [file pone.0106299.s001.tif]

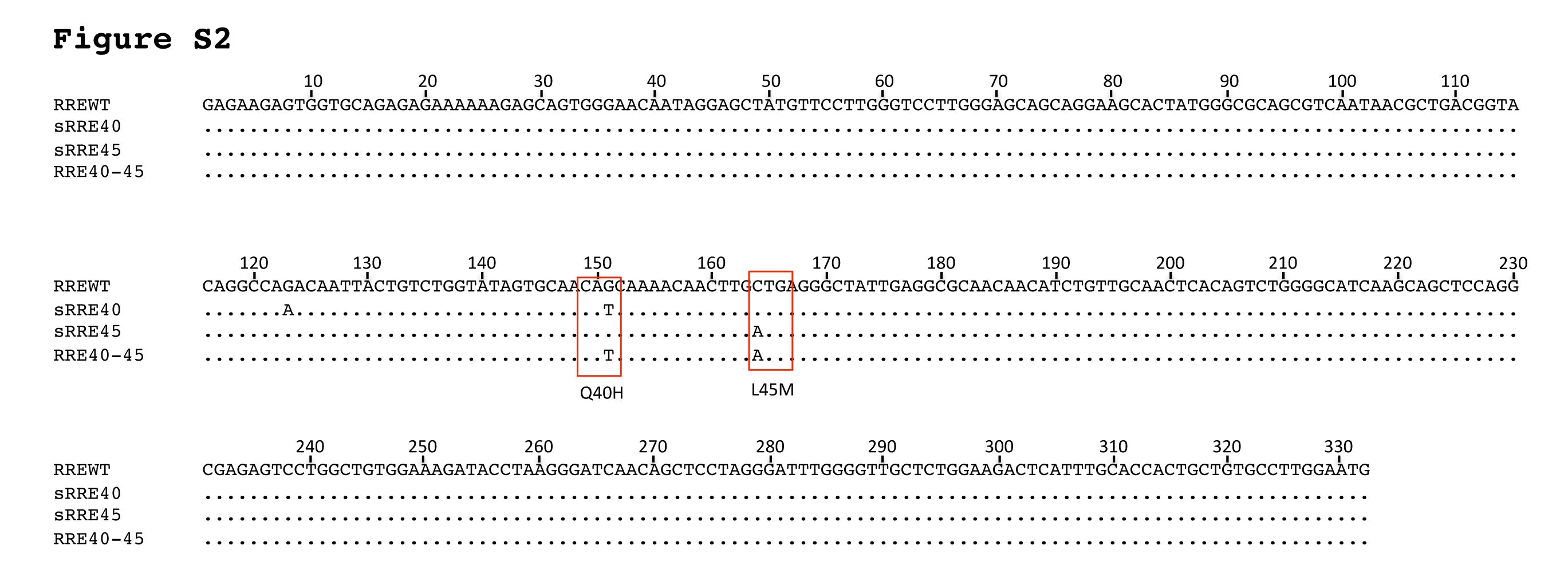

Supplement: Figure S2 — Multiple nucleotide alignment of RRE variants with nucleotide substitutions coding amino acids 40 and 45. The sRRE40 (Q40H), sRRE45 (L45M), and RREWT (40Q-45L) variants were created from a double mutant variant RRE40-45 (Q40H-L45M) by site-directed mutagenesis, cloned into a pPCR-Script vector and sequenced. Boxes highlight the nucleotides encoding the amino acids changed. (TIF) [file pone.0106299.s002.tif]

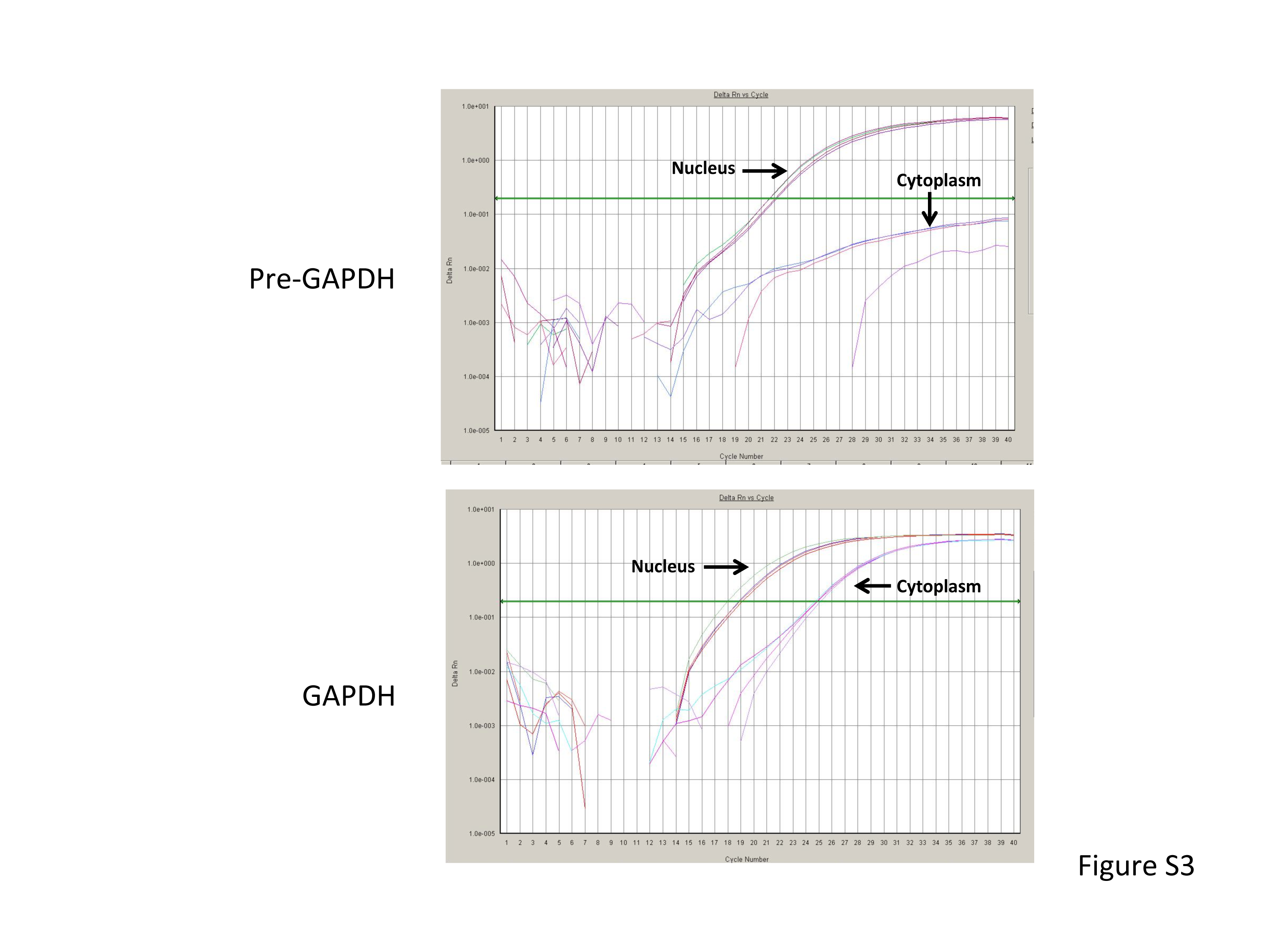

Supplement: Figure S3 — Real-Time RT-PCR of pre-GAPDH and GAPDH from nuclear and cytoplasmic fractions. Nuclear and cytoplasmic RNA fractions were isolated under standard conditions and were used to amplify the unspliced GAPDH (pre-GAPDH) and the total GAPDH with specific primers. (TIF) [file pone.0106299.s003.tif]
